# A retrospective database study comparing treatment outcomes and cost associated with choice of fixed-dose inhaled corticosteroid/long-acting β_2_-agonists for asthma maintenance treatment in Germany

**DOI:** 10.1111/j.1742-1241.2008.01895.x

**Published:** 2008-12

**Authors:** S Aballéa, S Cure, C Vogelmeier, A Wirén

**Affiliations:** 1 i3 InnovusUxbridge, UK; 2 Universitätsklinik Giessen und MarburgStandort Marburg, Marburg, Germany; 3 AstraZenecaLund, Sweden

## Abstract

**Aims:**

This retrospective, observational cohort study aimed to compare treatment outcomes and healthcare costs in the year after initiation of maintenance treatment with budesonide/formoterol or salmeterol/fluticasone in a German healthcare setting.

**Methods:**

Data on German asthma patients initiating treatment with budesonide/formoterol or salmeterol/fluticasone between June 2001 and June 2005 were obtained from the IMS Disease Analyzer database. The primary outcome was the probability of treatment success, defined according to short-acting β_2_-agonist prescriptions and switches or addition of controller medications, during the postindex year. A secondary definition of treatment success included hospitalisations and oral corticosteroid (OCS) prescriptions. Secondary outcomes included severe asthma exacerbations, defined as ≥1 OCS prescription, asthma-related hospitalisation and/or referral. The effect of treatment on costs was estimated using generalised linear models, adjusting for patient and physician characteristics.

**Results:**

There were no significant differences between the budesonide/formoterol (*n*=1456) and salmeterol/fluticasone (*n*=982) groups in disease severity markers in the pre-index year. Patients on budesonide/formoterol had a 44% greater probability of treatment success [odds ratio (OR): 1.44; p = 0.0003] according to the primary definition and a 26% greater probability (OR: 1.26; p = 0.0119) according to the secondary definition, fewer severe exacerbations (−33.4%; p = 0.0123) and fewer OCS prescriptions (−31.5%; p = 0.0082) compared with salmeterol/fluticasone, after controlling for baseline characteristics. Adjusting for covariates, budesonide/formoterol had a significant inverse relationship on asthma-related costs compared with salmeterol/fluticasone (−13.4%; p < 0.001). Total cost (asthma- and non-asthma-related costs) was 12.6% lower for budesonide/formoterol (p < 0.0001).

**Conclusion:**

This study suggests that for patients with chronic asthma in Germany, budesonide/formoterol rather than salmeterol/fluticasone had a higher likelihood of treatment success, and that budesonide/formoterol is the less costly option. Although the cohorts appeared to be well matched at baseline, the results should be interpreted with caution given the observational nature of the study.

**What’s known**

- Clinical studies have shown that, for patients with asthma uncontrolled by low-dose inhaled corticosteroids (ICS), maintenance treatment with fixed-dose combinations of an ICS and a long-acting β_2_-agonist (LABA) are effective in achieving asthma control and preventing exacerbations.
- Retrospective database studies have shown that combination products may increase compliance and treatment outcomes compared with separately administered ICS and LABA.
- Recent meta-analyses have indicated that the two most commonly used fixed-dose ICS/LABA combinations (budesonide/formoterol and salmeterol/fluticasone) may not have the same level of efficacy with regard to asthma-related hospitalisations/emergency room visits, the risk being greater with salmeterol/fluticasone.

**What new**

- This database analysis of fixed-dose ICS/LABA combinations adds to a growing body of evidence suggesting that there may be efficacy differences between budesonide/formoterol and salmeterol/fluticasone. The results suggest that, in a real-life German healthcare setting:
- Patients initiating treatment with budesonide/formoterol had an increased chance of treatment success and reduced exacerbations compared with salmeterol/fluticasone.
- Fixed-dose budesonide/formoterol is less costly than salmeterol/fluticasone for the treatment of chronic asthma.

## Introduction

Guidelines published by the Global Initiative for Asthma indicate that asthma control can and should be achieved and maintained in most patients by employing appropriate pharmacotherapy and minimising environmental stimuli (1,2). However, this goal is not always met and the majority of patients still remain uncontrolled. In a recent survey of over 2000 adults diagnosed with asthma in five European countries only 38% of treated patients were well controlled, and in Germany this figure was lower, at 26% (3). Currently, initial treatment generally consists of a fixed low dose of an inhaled corticosteroid (ICS) (200–500 μg beclomethasone dipropionate equivalent) with a short-acting β_2_-agonist (SABA) for symptom relief as needed (1,2). Clinical trials have demonstrated that, in patients in whom asthma is not controlled with low-dose ICS, the combination of an ICS and a long-acting β_2_-agonist (LABA) improves asthma control more effectively than higher doses of ICS alone (4–7). This combination is now the recommended maintenance therapy for patients stepping up from low-dose ICS (1,2). Fixed-dose combination inhalers have been developed, which offer improved convenience over ICS and LABA administered in separate devices and ensure better long-term compliance to anti-inflammatory therapy (8). Two previous database studies indicated that combination products improve both compliance and treatment outcomes compared with the concurrent administration of individual products via separate inhalers (9,10).

The two most commonly used fixed combination products, budesonide/formoterol (Symbicort®, AstraZeneca, Lund, Sweden) and salmeterol/fluticasone (Seretide™, GlaxoSmithKline, Uxbridge, UK or Viani™, GlaxoSmithKline), have both been shown to be highly effective in patients with persistent asthma that is uncontrolled with ICS alone (6,11–15). Randomised controlled studies in moderate-to-severe asthma have shown similar daily or weekly control and overall exacerbation rates for both combinations when used as fixed-dose maintenance therapy (16–18). Nevertheless, in the study by Kuna and colleagues and a recent meta-analysis of three clinical trials, including over 4000 patients, it was found that the risk or rate of exacerbations requiring hospitalisations/emergency treatments was lower with sustained fixed-dose budesonide/formoterol than with sustained fixed-dose salmeterol/fluticasone (18–20). This important efficacy difference was consistent in all three of the individual studies included in the analysis. Two recent meta-analyses also indicate that these two combinations may have different effects on asthma-related hospitalisations. In an analysis assessing the safety of formoterol administered in combination with an ICS in 18 double-blind clinical studies, fewer asthma-related hospitalisations and asthma-related serious adverse events were observed in the formoterol/ICS group compared with the ICS-alone control group (21). These results were independent of the ICS dose in the control group. In contrast, an analysis of 52 clinical studies of salmeterol showed no difference in asthma-related hospitalisations when salmeterol was added to an ICS compared with ICS alone (22). Thus, it appears that combination therapies may not have the same level of efficacy across all outcome measures important in asthma management.

It is not certain if the reduced risk of requiring hospitalisation/emergency treatment with budesonide/formoterol compared with salmeterol/fluticasone in meta-analyses of randomised controlled trials is likely to be seen in the normal clinical setting. In this case, database studies can provide valuable information on how these commonly used combination therapies affect cost-driving outcomes, such as exacerbations, in everyday practice. Such analyses are also important for establishing the overall relative cost-effectiveness of initiating treatment with combinations of budesonide/formoterol or salmeterol/fluticasone in asthma patients. The relative effectiveness and cost-effectiveness of initiating maintenance therapy with budesonide/formoterol and salmeterol/fluticasone under standard clinical conditions in Germany is not known. The aim of this retrospective database cohort study was to compare treatment outcomes and healthcare costs in the year after the initiation of maintenance treatment with budesonide/formoterol or salmeterol/fluticasone in a German healthcare setting.

## Methods

### Sources of data

This study was a retrospective database cohort study carried out using data obtained from the IMS Disease Analyzer database (http://www.imshealth.com). This is a database that captures real-life, longitudinal, anonymised data on patients and prescribers across Europe. In Germany, approximately 2000 physicians record data on 10 million patients. The data recorded include patient demographics, physician characteristics, prescriptions, hospital admissions and specialist referrals.

### Patient population

The cohorts identified for the study included patients who initiated maintenance treatment with budesonide/formoterol or salmeterol/fluticasone between June 2001 and June 2005. Budesonide/formoterol is reported to be effective when used as both maintenance and reliever therapy compared with traditional ICS/LABA combinations plus SABA (18,23,24). However, during the time period when the data used in this study was recorded, budesonide/formoterol was approved for use as maintenance treatment only. In this study, the index date was defined as the date of their first prescription of ICS/LABA combination. Eligible patients were over 12 years of age, had a diagnosis of asthma (ICD-10 codes J45–46) and continuous enrolment in the database from 12 months prior to the index date to 12 months after the index date (IMS data was collected between June 2000 and June 2006). Patients with diagnosed chronic obstructive pulmonary disease or use of ICS/LABA prior to the launch of budesonide/formoterol in Germany (June 2001) were excluded.

### Treatment outcomes

The predefined primary end-point was the probability of full treatment success vs. partial or no treatment success over 12 months following first prescription of an ICS/LABA combination therapy. Treatment success was defined *a priori* according to primary or secondary criteria as described in Table 1. All events were assumed to be asthma related if a code for asthma diagnosis (ICD-10 codes J45–J46) was recorded on the same date. In case there was no link to diagnosis for an event, an algorithm was used to define whether an event was related to asthma. Hospitalisations, referrals and oral corticosteroid (OCS) prescriptions were assumed to be related to asthma if they occurred within the 7 days before or after an event with a recorded diagnosis of asthma. Referrals associated with asthma were defined as all referrals to either a pneumologist or an allergologist.

**Table 1:** Definition of treatment success in the year following initiation of budesonide/formoterol or salmeterol/fluticasone therapy, according to primary and secondary criteria

Category	Primary success criteria	Secondary success criteria
Full success	Average SABA consumption of < 0.5 doses* per day and No addition of asthma medication (leukotriene antagonist,theophylline, omalizumab, fenoterol + ipratropium combination) between 10 and 52 weeks after index date or any switch to alternative ICS/LABA fixed combination or ICS + LABA	As for the primary definition and No asthma-related OCS prescription and No asthma-related referrals orhospitalisations
Partial success	An otherwise successfully treated patient who has an average SABA consumption between 0.51–2 doses per day	As for the primary definition and/or 1–2 OCS prescriptions and No asthma-related referrals orhospitalisation
No success	Average SABA consumption of more than two doses/day or Addition of asthma medication between 10 and 52 weeks after index date or switch to alternative ICS/LABA fixedcombination or ICS + LABA	As for the primary definition and/or > Two OCS prescriptions and/or At least one asthma-related referral orhospitalisation

* Expressed in dry powder inhaler equivalents. ICS, inhaled corticosteroid; LABA, long-acting β_2_-agonist; OCS, oral corticosteroid; SABA, short-acting β_2_-agonist.

Secondary outcomes included asthma exacerbations, SABA use, overall OCS prescriptions and asthma-related referrals and hospitalisations/emergency room visits. Asthma exacerbations were defined as any event (prescription, hospitalisation, sick note or referral) with a diagnosis of acute severe asthma status (ICD-10 code J46) or any asthma-related OCS prescription occurring within the 7 days before or the 7 days after an event with recorded diagnosis of asthma or acute lower respiratory tract infection (ICS-10 codes J45, J46, J22), or any referral or hospitalisation occurring within 7 days before or after an event with recorded diagnosis of asthma or acute lower respiratory tract infections. In cases where two or more acute exacerbations occurred within 7 days, it was counted as only one episode. The proportion of patients that did not renew their initial prescription was investigated as a measure of discontinuations.

### Cost analysis

The health economic evaluation was conducted from a third-party payer perspective and compared costs related to each therapy in terms of medications and other asthma-related healthcare resource use. Unit costs for asthma medications were estimated from producer sales prices using a program provided by IMS. For budesonide/formoterol and salmeterol/fluticasone, 2007/2008 prices were obtained from the Rote Liste (http://www.rote-liste.de). Costs for physician consultations and outpatient procedures were obtained from the Einheitbewertungsmaßstab schedules and costs for hospitalisations came from the German refined diagnosis-related groups (http://www.g-drg.de/) (Table 2).

**Table 2:** Unit costs

Type of cost		Unit	Cost, €
**Physician**			
	Patient age 12–59 years	Routine visit	6.75*
		Asthma-related visit	29.50*
	> 60 years	Routine visit	10.75*
		Asthma-related visit	33.50*
Allergologist		Referral	87.75*
Pneumologist†		Referral	57.20*
Hospitalisation associated with asthma		Hospital stay	1607.35‡
**G-DRG associated with asthma diagnosis**			
Bronchitis and bronchial asthma, > 55 years oldor with heavy complications (1619 cases)		Hospital stay (6.8 days)	2012.50§
Bronchitis and bronchial asthma, age6–59 years old and without heavycomplication (2118 cases)		Hospital stay (4.0 days)	1378.46§
Diseases/disturbances of respiratory organs,with artificial respiration > 24 h, withoutcomplication (6 cases)		Hospital stay (10.2 days)	5651.31§

* Unit costs obtained from EBM schedules, assuming a unit cost of €0.05 for each EBM point.

† It was assumed that two spirographies and one body plethysmography were conducted for all referrals.

‡ An estimated average cost calculated from the three G-DRG associated with a possible main diagnosis of asthma or acute exacerbation. This weighted average cost was calculated based on the number of cases associated with one asthma diagnosis and a co-payment of €10 per hospital day was applied.

§ Unit costs obtained from G-DRG (http://www.g-drg.de/). G-DRG, German refined diagnosis-related groups; EBM, Einheitbewertungsmaßstab.

### Statistical analysis

Logistic regression was used to estimate the association between treatment group and treatment success, adjusting for patient characteristics. Treatment success was modelled as a dichotomic variable, grouping the categories ‘partial success’ and ‘no success’ together. Potential covariates included disease severity according to treatment history, age and insurance status, and centre characteristics according to the physician and the specialty of the lead physician. Covariates that were not statistically significant at the 5% level were removed. Renewal rates of ICS/LABA prescriptions were assessed as a measure of treatment persistence. Generalised linear models were used to estimate the effect of treatment on severe exacerbations, resource use and costs, adjusting for patient and physician characteristics. The choice of statistical distribution was based on goodness-of-fit statistics, which lead to the application of lognormal and gamma distributions, depending on the type of resource or cost modelled.

## Results

### Patients

**Figure 1:**
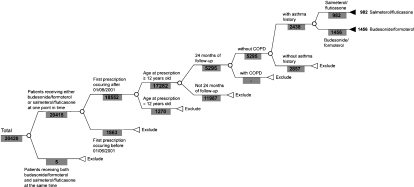
Selection of patients from the IMS Disease Analyzer database

Among the 2438 patients who met the study inclusion criteria, 1456 were treated with budesonide/formoterol (administered via Turbuhaler) and 982 were treated with salmeterol/fluticasone (administered via Diskus in the majority of patients). These patients represented 7.1% and 4.8% of the database respectively (Figure 1). The majority of exclusions were due to patients not fulfilling 24 months of follow-up or having no history of asthma. Patient characteristics are summarised in Table 3. The mean age was approximately 48 years in both groups and there were no significant differences between groups in markers of disease severity in the pre-index year, based on prescriptions of asthma medication, referrals (p = 0.2829) or hospitalisations (p = 0.7764). SABA prescriptions in the pre-index year were recorded in 41.2% and 39.8% of patients in the salmeterol/fluticasone and budesonide/formoterol groups, respectively, at an average of approximately 1.8 inhalations per day [dry power inhaler (DPI) equivalent]. There was no statistically significant difference in reported comorbidities between groups (rhinitis, p = 0.0867 and gastro-oesophageal reflux disease, p = 0.9764).

**Table 3:** Characteristics of patients initiating treatment with budesonide/formoterol or salmeterol/fluticasone

Characteristic	SAL/FLU group (*n*=982)	BUD/FORM group (*n*=1456)	p-value†
Females, %	55.0	57.2	0.278
Mean age, years	47.6	48.4	0.32
**Insurance status, %**			
Common medical insurance plan	28.1	28.9	0.0008
Other	11.2	11.3	
Company health insurance fund	16.8	20.1	
Substitute sickness insurance society	29.5	30.7	
Private health insurance	14.4	9.0	
**Lead physician, %**			
General practice	72.0	76.2	0.0022
Internal medicine	20.9	19.8	
Specialist (other)	7.1	4.1	
**Comorbidities, %**			
Rhinitis	15.6	13.1	0.0867
Gastro-oesophageal reflux disease	3.9	3.9	0.9764
**Events in pre-index year, %**			
At least one referral related to asthma	10.6	9.3	0.2829
At least one hospitalisation related to asthma	0.7	0.6	0.7764
Prescriptions in pre-index year*, %			
SABA	41.2	39.8	0.4663
ICS	28.2	26.0	0.2344
OCS	8.6	6.5	0.0596
LABA	13.5	13.9	0.8165
ICS + LABA non-fixed combination	10.1	8.2	0.1053

* Proportion of patients with at least one prescription. This reflects the lowest possible proportion as patients could have received prescriptions at another practice not reporting to the IMS Disease Analyzer. Note, because of rounding percentages, these may not always add up to 100%.

† p-values for association with type of practice and insurance are based on independence chi-square tests. All others are *t*-tests. BUD/FORM, budesonide/formoterol; ICS, inhaled corticosteroid; LABA, long-acting β_2_-agonist; OCS, oral corticosteroid; SABA, short-acting β_2_-agonist; SAL/FLU, salmeterol/fluticasone.

In both groups, the majority of patients were followed at a general practitioner (GP)-lead clinic and the minority were followed at a specialist-lead practice. However, comparison of the two groups showed a higher proportion of patients at GP-lead clinics in the budesonide/formoterol group and a higher proportion of patients followed by specialists in the salmeterol/fluticasone group. These differences resulted in a statistically significant association between treatment group and type of practice (p = 0.0022). Insurance status also differed significantly between treatment groups (p = 0.0008). More patients in the budesonide/formoterol group had a company health insurance fund, while more patients in the salmeterol/fluticasone group had private insurance.

### Treatment success

**Figure 2:**
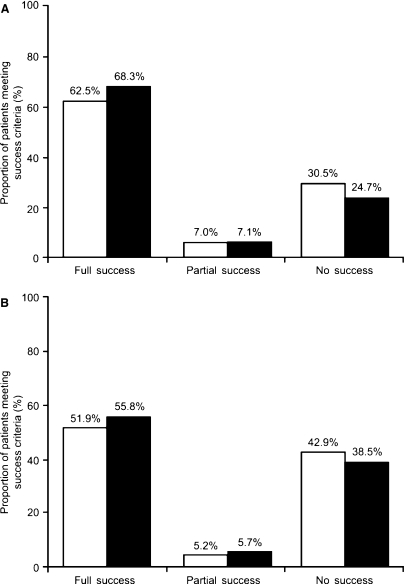
Proportions of salmeterol/fluticasone- (white bars) or budesonide/formoterol- (black bars) treated patients meeting the criteria for primary (A) or secondary (B) treatment success. p-values calculated via chi-squared test of independence between treatment success and treatment group, for primary success, p = 0.0060 and for secondary success, p = 0.0928

A higher proportion of patients in the budesonide/formoterol cohort met the criteria for primary and secondary treatment success compared with the salmeterol/fluticasone cohort (unadjusted analysis; Figure 2). As expected, the proportion of successfully treated patients for both budesonide/formoterol and salmeterol/fluticasone was lower when applying the secondary definition, which included OCS use and referrals (Figure 2). For both definitions of treatment success, a larger proportion of patients in the salmeterol/fluticasone group, compared with the budesonide/formoterol group, failed to meet each of the subcriteria for full success (37.5% vs. 31.7%, Table 4). The most common reasons for treatment failure in both groups were addition of another asthma medication, SABA use at > 0.5 doses/day (DPI equivalent) and two or more asthma-related referrals (Table 4).

**Table 4:** Reasons for failure to meet primary or secondary criteria for treatment success

Reason (% of all patients)	SAL/FLU group (*n*=982)	BUD/FORM group (*n*=1456)
**SABA use**		
> 0.5 to ≤ 2 doses/day	15.7	15.2
> 2 doses/day	8.8	7.8
Addition of other asthma medication* between week 10 andweek 52 after index date	25.4	20.2
Switch from ICS + LABA combination to another	7.0	3.6
**OCS prescriptions**		
1–2 prescription	5.2	3.8
> 2 prescriptions	2.0	0.9
**Referrals related to asthma**		
1 referral	5.7	3.9
> 2 referrals	9.9	9.0
**Hospitalisation related to asthma**		
1 hospitalisation	1.1	0.3
> 2 hospitalisations	0.2	0.2

* Leukotriene antagonist, IgE (omalizumab), anticholinergic (ipratropium, ipratropium + fenoterol combination), theophylline, combination cromolyn/reproterol. Not more than 11% of failures in either group were associated with any one medication. BUD/FORM, budesonide/formoterol; ICS, inhaled corticosteroid; LABA, long-acting β_2_-agonist; OCS, oral corticosteroid; SABA, short-acting β_2_-agonist; SAL/FLU, salmeterol/fluticasone.

**Figure 3:**
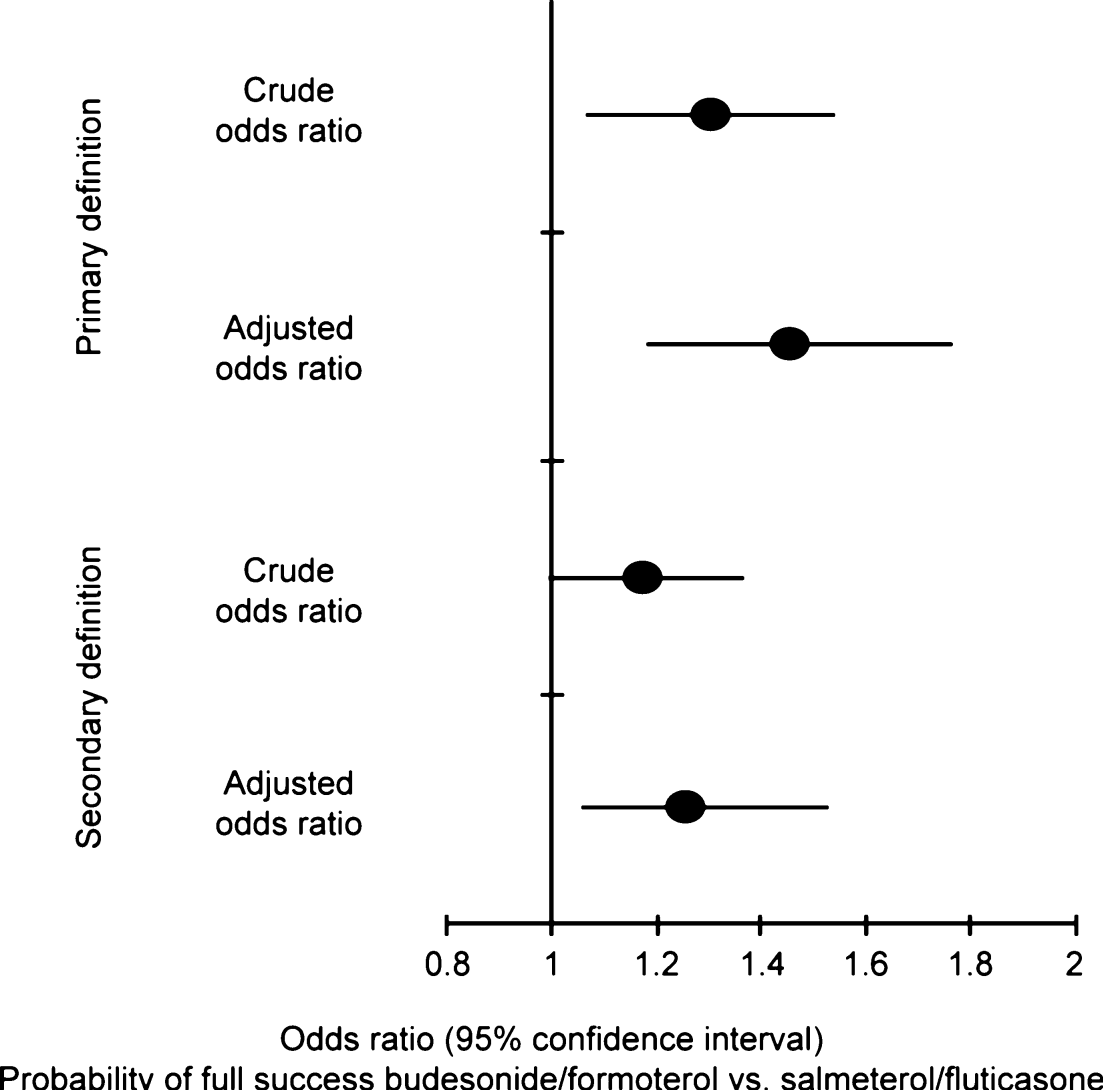
Relative probability of full treatment success. Values to the right of the vertical line indicate a higher probability of full treatment success with budesonide/formoterol compared with salmeterol/fluticasone. The filled circles represent the point estimate for the odds ratios (OR) and the horizontal lines the range of the confidence intervals. The regression models used to derive the adjusted OR included pre-index inhaled corticosteroid (ICS), long-acting β_2_-agonist (LABA) and short-acting β_2_-agonist (SABA) use for the primary definition and pre-index ICS, LABA and SABA use, age and physician speciality for the secondary definition

In the logistic regression model adjusted for disease severity and pre-index SABA use, the odds for achieving the primary definition of ‘full success’ vs. ‘partial or no success’ increased by 44% with budesonide/formoterol [odds ratio (OR): 1.44; p = 0.0003, Figure 3]. Patients treated with budesonide/formoterol also had a greater probability of treatment success when applying the secondary definition (OR: 1.26; p = 0.0119).

Subgroup analyses were conducted according to prescription renewal of ICS/LABA combination treatment. The logistic regression model for the primary definition of treatment success showed a significant treatment effect with budesonide/formoterol for the subgroup of the 1169 patients who did not renew their initial prescription (OR: 1.422; p = 0.0333) and for the 1269 patients with at least two prescriptions (OR: 1.426; p = 0.0064). For the 586 patients with a prescription renewal within 4 months of index date the OR was 1.214, which did not reach statistical significance (p = 0.3011).

### Other treatment outcomes

A significantly higher number of severe exacerbations was observed among patients treated with salmeterol/fluticasone compared with budesonide/formoterol in the unadjusted analysis (0.1955 vs. 0.103 episodes per patient per year; p = 0.0018; Table 5). Linear regression analysis (adjusted for pre-index ICS, LABA and SABA use, gender and numbers of exacerbations, OCS prescriptions, referrals and hospitalisations over the pre-index period) indicated that budesonide/formoterol reduced the number of severe exacerbations by 33.4% compared with salmeterol/fluticasone (p = 0.0123). There was no statistically significant difference in the mean numbers of asthma-related referrals or hospitalisations between the budesonide/formoterol and the salmeterol/fluticasone groups and mean SABA use did not differ between groups in the postindex year. However, overall OCS prescriptions were more frequent in the salmeterol/fluticasone-treated patients compared with the budesonide/formoterol-treated patients. Almost half of the patients in both the salmeterol/fluticasone and the budesonide/formoterol groups (45% and 49.4% respectively) did not renew the prescription of their initial ICS/LABA combination.

**Table 5:** Secondary treatment outcomes in the post-index year

			Relative difference (BUD/FORM vs. SAL/FLU)		
Outcomes	SAL/FLU group (*n*=982)	BUD/FORM group (*n*=1456)	Crude (%)	Adjusted* (%)	p-value*
Asthma exacerbations (mean episodes/patient)	0.20	0.10	−47.3	−33.4	0.0123
SABA use (mean number of doses/patient/day)	0.60	0.52	−13.7	−8.7	0.2297
OCS (mean prescriptions/patient)	0.30	0.17	−47.3	−31.5	0.0082
Asthma-related referrals (mean number/patient)	0.19	0.18	−9.5	−9.5	0.4358
Asthma-related hospitalisations (mean number/patient)	0.021	0.012	−45.4	−2.9	0.7228

* Adjusted for pre-index inhaled corticosteroid, long-acting β_2_-agonist and short-acting β_2_-agonist (SABA) use, gender and numbers of exacerbations, oral corticosteroid (OCS) prescriptions, referrals and hospitalisations over the pre-index period. BUD/FORM, budesonide/formoterol; SAL/FLU, salmeterol/fluticasone.

### Costs

**Figure 4:**
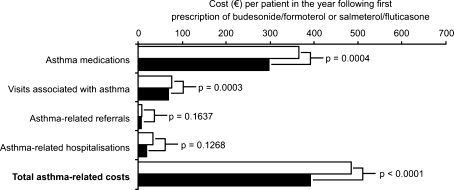
Crude comparison of costs in the postindex year between salmeterol/fluticasone- (white bars) and budesonide/formoterol- (black bars) treated patients. p-values were derived using an unequal variance *t*-test

Total mean asthma-related costs were significantly greater for the salmeterol/fluticasone group compared with the budesonide/formoterol group (Figure 4). The biggest differences were seen for asthma-related medications and asthma-related visits, for which costs were significantly greater for the salmeterol/fluticasone group than the budesonide/formoterol group. Mean costs associated with asthma-related referrals and hospitalisations were not significantly different between treatment groups.

A generalised linear model using a log-normal distribution was used to estimate the effect of treatment on costs. When adjusting for covariates, budesonide/formoterol reduced total asthma-related costs (−13.4%: p < 0.001) compared with salmeterol/fluticasone. Total cost, including both asthma-related (medication, visits, referrals and hospitalisations) and non-asthma-related (medications and visits) costs, was 17.1% lower for budesonide/formoterol compared with salmeterol/fluticasone in the crude comparison (683.57€ vs. 824.67€; p = 0.0001) and 12.6% lower in the adjusted analysis (p < 0.0001).

## Discussion

Clinical studies have shown that for patients with asthma uncontrolled by low-dose ICS treatment, fixed-dose combinations of an ICS and a LABA are more effective than either ICS alone or separately administered ICS and LABA (8–10). This database analysis of fixed-dose ICS/LABA combinations adds to a growing body of evidence suggesting that there may be efficacy differences between budesonide/formoterol and salmeterol/fluticasone across certain cost-driving outcomes. The results suggest that, in a German healthcare setting, better outcomes can be achieved at a lower overall cost if patients are treated with budesonide/formoterol instead of salmeterol/fluticasone.

The economic cost of asthma is considerable both in terms of direct medical costs (such as hospital admissions and cost of pharmaceuticals) and indirect costs (such as time lost from work and premature death). Ineffective management of asthma can increase these costs, and the overall cost-effectiveness of new therapies is an important consideration. Economic analyses in Sweden have shown that combining budesonide and formoterol in one device may be cost-effective compared with the two agents administered separately (25,26). This study is the first of its kind to compare the cost-effectiveness of budesonide/formoterol and salmeterol/fluticasone using a German database analysis. The analysis indicates that, relative to the use of salmeterol/fluticasone, budesonide/formoterol treatment is less costly and is associated with a greater rate of treatment success in the German healthcare setting. The difference in cost appeared to be driven by fewer physician visits and lower expenditure on medications in the treatment practices where budesonide/formoterol was commonly used than in the practices where salmeterol/fluticasone was used. In database surveys, both the clinician and the treatment are likely to influence outcomes. Of note, in this analysis there were some differences between the two treatment groups in terms of lead physician type and insurance status. A higher proportion of patients in the budesonide/formoterol group were followed at a GP-lead clinic, while a higher proportion of the salmeterol/fluticasone group were followed at a specialist-lead clinic. Although statistically significant, these differences were small and have been controlled for through the use of regression analyses.

The fact that a high proportion of patients did not continue treatment with ICS/LABA for the whole study period, particularly in the budesonide/formoterol group, commands caution in interpreting the results. However, the effect of budesonide/formoterol on treatment success in the subgroup of patients who did not renew their initial prescription (OR: 1.422; p = 0.0333) was similar to the effect estimated in the whole sample, using logistic regression. Among patients with at least one prescription renewal within 4 months of the index date, the probability of treatment success was also higher for those treated with budesonide/formoterol, although the corresponding effect was not statistically significant. The ICS/LABA prescription renewal rates observed in this analysis are similar to those reported in a pharmacy database study investigating salmeterol/fluticasone adherence and persistence in 5504 patients in the USA (27). More than half the patients filled a 30-day prescription only once over a 1-year interval, suggesting that adherence to ICS/LABA combinations may be considerably lower than those reported in clinical trials. Evidence also indicates that, in ‘real-life’, adherence to asthma therapy is greater among patients using fixed-dose combination inhalers compared with those prescribed separate ICS and LABA inhalers (8).

As with all database studies there are several other limitations not usually encountered in controlled clinical trials that must be considered. Lack of follow-up between practices and the absence of any record of medications prescribed by specialists could lead to underestimation of prescriptions. However, this is unlikely to account for the differences observed between treatment groups in this comparative study as pre-index referrals and asthma-related prescriptions were similar between the two groups. Missing diagnoses and the use of algorithms to identify asthma-related events may also affect the results; however, this is also unlikely to have resulted in any significant bias in this study because proportions of missing diagnoses between treatment groups were similar. Of note, the probability of success was significantly higher in the budesonide/formoterol group in analyses using other variants of the definition of treatment success, taking into account events not related to asthma (results not reported). Furthermore, costs were lower in the budesonide/formoterol group regardless of whether costs unrelated to asthma, including costs for other medications and non-asthma-related physician visits, were included or not.

Two key objectives listed in guidelines for the successful management of chronic asthma are to achieve and maintain control of symptoms and to reduce or prevent asthma exacerbations. The results of this study suggest that in the German healthcare setting these goals are achieved more often in patients prescribed budesonide/formoterol than in patients prescribed salmeterol/fluticasone, and that budesonide/formoterol appears to represent a more cost-effective option based on the available evidence. Although the cohorts appeared to be well matched at baseline, the results should be interpreted with caution given the observational nature of the study.
